# The Effect of an Enriched Sport Program on Children’s Executive Functions: The ESA Program

**DOI:** 10.3389/fpsyg.2020.00657

**Published:** 2020-04-28

**Authors:** Ambra Gentile, Stefano Boca, Fatma Neşe Şahin, Özkan Güler, Simona Pajaujiene, Vinga Indriuniene, Yolanda Demetriou, David Sturm, Manuel Gómez-López, Antonino Bianco, Marianna Alesi

**Affiliations:** ^1^Ph.D. Program in Health Promotion and Cognitive Sciences, University of Palermo, Palermo, Italy; ^2^Department of Psychology, Educational Sciences and Human Movement, University of Palermo, Palermo, Italy; ^3^Faculty of Sports Science, Ankara University, Ankara, Turkey; ^4^Department of Coaching Science, Lithuanian Sports University, Kaunas, Lithuania; ^5^Department of Physical and Social Education, Lithuanian Sports University, Kaunas, Lithuania; ^6^Department of Sport and Health Sciences, Technical University of Munich, Munich, Germany; ^7^Department of Physical Activity and Sport, Faculty of Sports Sciences, University of Murcia, Murcia, Spain

**Keywords:** training program, high-order cognitive abilities, cognitive flexibility, inhibitory control, physical education class, working memory

## Abstract

**Purpose:**

The effects of physical exercise on executive functions (EFs) are well-documented. EFs are involved in daily activities, and their development determines the quality of people’s future life, both in terms of mental health and quality of life. The purpose of the current paper is to evaluate the effects of a physical education program, elaborated within the Enriched Sports Activity Program (ESA Program), an Erasmus + Project, on EFs, namely, visuospatial working memory, inhibitory control, cognitive flexibility, and task switching.

**Method:**

Data were collected on November 2017 (t_1_) and May 2018 (t_2_). At t_1_, a sample of 357 children from four European countries (Italy, Germany, Lithuania, and Turkey) performed a cognitive test battery made up of Digit Span Forward/Backward, Stroop Task, and Trail Making Test (TMT), whose order was randomized. From November until May, classrooms from the experimental group followed the ESA Program, while classrooms from the control continued with the ordinary physical education class. At t_2_, children from both experimental and control groups performed again the cognitive battery.

**Result:**

The repeated measures ANOVA showed a significant effect of the ESA Program on the TMT B and on Digit Span Backward, but no significant effects were found on Digit Span Forward and Stroop Task.

**Conclusion:**

The introduction of a sport program enriched with cognitive stimuli has beneficial effects for children working memory and cognitive flexibility.

## Introduction

In the past decades, cognition and exercise have been considered as separate domains and, for this reason, were treated independently (Diamond, 2000). In recent years, a close link between physical exercise and cognitive abilities has been recognized (Colcombe and Kramer, 2003), specifically for what concerns the influence of physical exercise on executive functions (EFs).

Many hypotheses have been provided for explaining this improvement as the increase of the catecholamine levels (Chmura et al., 1994; Verburgh et al., 2014), which is linked to prefrontal cortex activity as well as executive functioning (Mehren et al., 2019), or the increase of cerebral blood flow (CBF) due to exercise (Verburgh et al., 2014). During childhood, prefrontal cortex activation should be more dynamic, and brain plasticity should encourage a permanent improvement in cognitive functioning (Khan and Hillman, 2014; Erickson et al., 2015.

EFs relate to a set of cognitive abilities that supervise the information processing for the implementation of goal-directed actions and that require a certain amount of memory, attention, inhibition, and self-control (Eslinger, 1996; Best, 2010; Davis et al., 2011). Especially for children, EFs are involved in the learning process (Alloway and Alloway, 2010); therefore, they are considered crucial for successful performances at school and the development of academic skills (Nayfeld et al., 2013). A study of Visu-Petra et al. (2011) revealed that EFs significantly predict children’s academic achievements. More specifically, working memory tasks forecast their performance in math tests, while the inhibition task is positively associated with the general semester grade. Haapala (2013) undertook a systematic literature review from 1966 to 2011 on physical activity, academic performance, and cognition in children and adolescents, providing evidences which showed positive effects of 14- to 36-week physical exercise training on mathematical, reading, and language achievement scores.

However, studies hypothesizing a connection between EFs and specific academic skills (e.g., math skills) found mixed results ranging from a positive influence of physical exercise to none on academic performances. A meta-analysis by de Greeff et al. (2018) found positive moderating effects of inhibition in the relationship between acute exercise and academic achievement, while no moderating effects were found concerning working memory and cognitive flexibility. Moreover, for what concerns physical activity programs, the meta-analysis revealed a significant moderating effect of working memory and cognitive flexibility, while no effects were detected concerning inhibition and planning. These inconsistent results, probably, are due to methodological difficulties in the EFs measurement that are, in turn, related to the complex conceptualization of the construct (Van der Ven et al., 2012). Indeed, the conceptualization of EFs has changed throughout time. In the past, the EF system was considered a unitary structure, while today, researchers have recognized the existence of different, distinguished, but interrelated functions (Miyake et al., 2000).

Hence, a good development in EF is crucial for youth, since problems encountered in EFs during childhood are related to problems in terms of health, social status, and quality of life later on in adult life (Jacka et al., 2011; Diamond, 2012). For this reason, EFs have been related to children with several health issues, as attention deficit hyperactivity disorder (ADHD) (Kempton et al., 1999; Holmes et al., 2010; Ziereis and Jansen, 2015), autism (McEvoy et al., 1993; Gilotty et al., 2002; Rosenthal et al., 2013), and brain injuries (McCarthy et al., 2005; Kesler et al., 2011).

A significant improvement in children’s cognitive abilities due to physical exercise has been found by Sibley and Etnier (2003). Specifically, physical activity seems to have positive effects on EF inhibitory function, planning and problem solving, cognitive flexibility, and visuospatial attention (Bidzan-Bluma and Lipowska, 2018). To what concerns the amount and frequency of physical exercise, a meta-analysis by Verburgh et al. (2014) has shown that acute physical exercise, which is a single bout exercise whose duration lasts from 10 to 40 min, has an overall effect on EFs, while no effects were detected concerning chronic exercise; that is, an exercise program lasting between 6 and 30 weeks. According to the authors, two main explanations may be provided: on the one side, chronic exercise has a smaller positive effect on cognitive functioning if compared to the acute exercise; on the other side, the studies included in the meta-analysis might not be suitable in terms of intensity, frequency, and duration. Moreover, while in acute exercise studies, the cognitive assessment took place immediately after the physical exercise, no information was provided concerning chronic exercise. Concerning the age, for children from 6 to 12 years, the authors found a moderate improvement of cognitive function after acute exercise. The importance of physical exercise for children EFs is crucial especially for low performer children who register a greater improvement compared to normal performer children (Diamond, 2012).

Considering that physical exercise has beneficial effects on children’s cognition, increasing attempts have been done to implement physical activity programs enriched with cognitive challenges (Hillman et al., 2008). Tuckman and Hinkle (1986) compared children’s physical and cognitive performance in a specific running program and an ordinary physical education class, finding that runners also became more creative than children in the ordinary class condition. Concerning this topic, Memmert (2006) confirmed that a sport enriched program can improve sport-related creative thinking, since children learn to perform successful behavior and to act it through creative motor functions. Davis et al. (2007) tested a physical activity program for obese children, analyzing the potential benefits of the program on EFs, and finding positive effects related to planning ability in high dose of exercise condition (40 min/day) compared to the no-exercise control condition.

The success of a sports program depends on its structure and its features. Diamond (2012) maintains that an effective aerobic training program can reduce disparities in EFs produced by differences in social status and predicts further academic success. Nevertheless, the improvement in one EF can be transferred to another, given the abovementioned multidimensional structure of the EF system.

The forecasted physical activity should also challenge children throughout the program. From a motivational point of view (Deci and Ryan, 2008), if kids are not pushed to do better, they stop improving, and, on the other side, if the activity does not become challenging, children get bored and abandon the program. Finally, single bouts of aerobic activity have produced the best results on EFs, but programs that last over time show smaller effects, thus Diamond (2012) suggests to create combined programs of physical training and character development activities.

Considering these findings, the current paper analyzes the effect of Enriched Sports Activity Program (ESA Program) on children’s EFs. The ESA Program is an enriched physical exercise protocol experimented within the Erasmus + Project *Enriched Sports Activity Program* (ESA Program; Agreement Nr.: Sport-579661-EPP-1-2016-2-IT-SPO-SCP). The project, which lasted 3 years, aimed at enhancing social inclusion, equal opportunity, and psychosocial well-being in children through an enriched protocol that introduced physical exercises able to stimulate cognitive growth (Alesi et al., 2017). The exercises were modified to stimulate the three core EFs, namely, inhibitory control, the ability to ignore one stimulus and concentrate on another; working memory, the goal-directed ability to monitor and manipulate mental representations stored in working memory; and task shifting, the ability to consciously switch from one task to another (Miyake et al., 2000; Diamond, 2013). The program was articulated into 27 units, divided into a 10-min baseline phase and a 15-min stimulation phase. The program is innovative since it standardized the warm-up session through the introduction of cognitive stimuli within exercises relating to several sports activities.

Considering the results retrieved from literature, the physical exercise protocol foreseen by the ESA Program should enhance EF performance in inhibitory control, working memory, and task shifting. Given the developmental phase of participants, ranging from 7 to 14 years, we expect a general improvement of all children’s EFs, but a particular improvement in children who have followed the ESA protocol.

## Materials and Methods

Data collection took place in the first months of the school year in November (t_1_) and in May (t_2_), at the end of the same school year, within four different European countries (Italy, Lithuania, Turkey, and Germany) (Table 1). Four hundred twenty-two children were included in the sample, but 65 children did not complete the final evaluation. Thus, the research sample consisted of 357 children (48% males, 52% females) whose age was comprised between 7 and 14 years old (mean age = 9.55, SD = 1.77). The study was carried out according to the Helsinki Declaration (Hong Kong revision, September 1989). It also received permission from the Lithuanian Sports University’s Research Ethics Committee in Social Sciences with approval No 579661-EPP-1-2016-2-IT-SPO-SCP (2018-02-05).

**TABLE 1:** Selected participants per country.

Country	N	Intervention	Control
Italy	164	77	87
Lithuania	93	56	37
Turkey	80	40	40
Germany	85	38	36

### Procedure

Cognitive data collection at t_1_ took place within four European countries (Italy, Lithuania, Germany, and Turkey). After parents’ signature of the consent form, children school classes were split in experimental and control groups. Children from both experimental group (ESA group) and control group completed a battery made up of three neuropsychological tasks derived by the Inquisit Lab platform: the Color Word Stroop Task (Stroop, 1935), the Trail Making Test (TMT) (Reitan, 1958), and the Digit Span Test (Lumley and Calhoon, 1934). One of the experimenters guided the children during the assessment, explaining the tasks they had to complete. Cognitive tasks were presented in a random order, and the data collection lasted about 30 min per respondent.

Children following the ESA Program completed 27 training units during physical education class, while children from the control group followed ordinary physical education class. At t_2_, corresponding to the end of the school year, children repeated the same cognitive assessment in the same order presented at t_1_.

### Measures

#### Executive Functions

The following three EFs were measured: working memory, inhibitory control, cognitive flexibility and task-switching ability (Sánchez-Cubillo et al., 2009). For this purpose, Inquisit five by Millisecond© was employed, using the libraries available on the Millisecond© website. These scripts implemented the classic Stroop Task, TMT, and Digit Span Test with keyboard inputs.

##### Digit span forward/backward

Digit Span assesses working memory (Lumley and Calhoon, 1934; Woods et al., 2011). In the visual version, numeric sequences appear on the screen, and participants have to recall them (both in a forward and in a backward manner) by selecting with the mouse the digits from a circle of digits. Depending on performance, participants move up a level or down a level, and the assessment is over after 14 trials. The whole task lasts 15 min. The number of recalled digits before two consecutive errors was taken into consideration for data analysis. Scores are computed counting the number of recalled digits in the presented order and the number of recalled digits in the reversed order.

##### Stroop task

The Stroop Task is designed for assessing inhibitory control (Stroop, 1935). Participants are showed on the computer screen words written in four colors, such as red, green, blue, black. The task is made up of three conditions: in the congruent task (W), participants see some words on the screen and they have to indicate in which color the word is written that is congruent with its meaning, e.g., “red” word printed in red color. In the incongruent task (CW), participants have to indicate in which color the word is written and ignoring its meaning, e.g., “blue” word printed in red color. In the control condition (C), participants see some blocks on the screen, e.g., colored rectangles, and they have to indicate the color. Total trials are 84 derived by: 4 colors (red, green, blue, black) × 3 color-stimulus congruency (congruent, incongruent, control) × 7 repetitions. In the current study, the interference score was calculated considering the logarithmic difference between inhibitory reaction time and control reaction time (MacLeod, 1991).

##### Trail making test

The TMT is a test developed for assessing cognitive flexibility and task switching (Reitan, 1958). In the A version, participants have to link numbers in increasing order, while in the B version, participants have to connect a number and a letter in an increasing way alternatively (i.e., 1-A-2-B-3-C). The trails were 4:1. Only numbers from 1 to 5; 2. only numbers from 1 to 25; 3. numbers and letters from 1A to 5; 4. numbers and letters from 1A to 13. For the calculation of the scores, only the B version completion time was used since it is more sensitive to cognitive flexibility skill (Kortte et al., 2002).

#### Enriched Sport Program

*ESA Program.* The implementation lasted 14 consecutive weeks in the school and the sports center context. It involved children from 7 to 14 years who already practiced sport. The protocol aimed to enrich the warm-up of regular sports activities with cognitive stimuli (inhibition, working memory, task shifting) for improving children’s EF. The program consisted of 27 units lasting 25 min. The unit was made up of a baseline phase and stimulation phase, and it was obtained through a combination of two features: cognitive stimulus and movement domain. The construct validity was assessed only qualitatively: four experts from psychology and sport science rated the extent to which an exercise could stimulate a specific cognitive function. The discrepancies were solved through discussion.

The cognitive stimuli for the stimulation phase could involve inhibitory control, working memory, or task shifting. In the activity stimulating inhibitory control, the coach’s verbal command for an exercise corresponded to the execution of another movement previously associated. For example, the verbal command “Skip-ahead” corresponded to “Kicked-ahead” movement, and the command “Fore-foot gait-ahead” corresponded to “Rear-foot gait-ahead.” The stimulation of working memory occurred through the explanation of a series of exercises that children had to perform in a reverse order. For example, the oral command “Balance on the line-ahead/behind” corresponded to the performance “Balance on the line-behind/ahead.” Concerning the task shifting stimulation, a circuit of exercises was created, and each child had to perform a specific exercise that was different from the others’ one. When the instructor whistled, children had to switch to the exercise that the kid ahead was performing, until all of them had performed all the exercises in the circuit.

**FIGURE 1:**
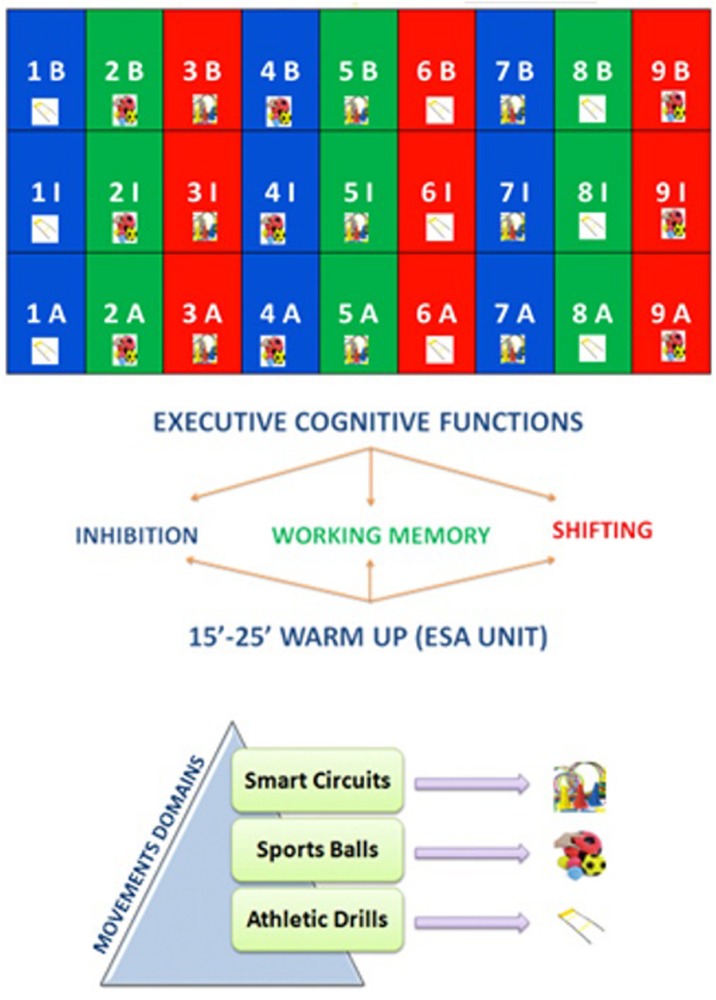
The Enriched Sports Activity (ESA) Program Protocol.

All the three domains started with a beginning level (B), followed by an intermediate level (I), and finishing with an advanced level (A). The design of the protocol was the following: the first nine units concerned the beginner level of exercise, whose domain was alternatively athletic drill, then sports ball, and finally smart circuits, and whose stimulation concerned alternatively working memory, inhibition, and task shifting. The same structure was kept for intermediate level and advanced level (Figure 1).

A series of coaches’ guidelines video tutorials were recorded to maximize the protocol standardization across the European administrators.

### Data Analysis

Descriptive statistics was performed on the sample, including height and weight (Tables 2, 3). Afterward, for evaluating the effects of the ESA Program on children’s EFs, a repeated measures ANOVA model with Time × Group comparisons was performed. Specifically, the cognitive scores at the beginning and the end of the school year were compared, separating the general effect of improvement from the one induced by the ESA Program. The age of the participants was included as a covariate since the effects of physical exercise enhance executive function performance at different levels across ages (Ludyga et al., 2016).

**TABLE 2:** Distribution of height in different age ranges.

	Height (cm)	
Age	Mean	SD
7–8 years	131.0	6.19
9–10 years	140.0	8.62
11–12 years	154.0	9.58
13–14 years	165.0	5.42

**TABLE 3:** Distribution of weight in different age ranges.

	Weight (kg)	
Age	Mean	SD
7–8 years	30.0	6.66
9–10 years	36.9	7.14
11–12 years	43.2	7.25
13–14 years	53.9	6.20

## Results

**FIGURE 2:**
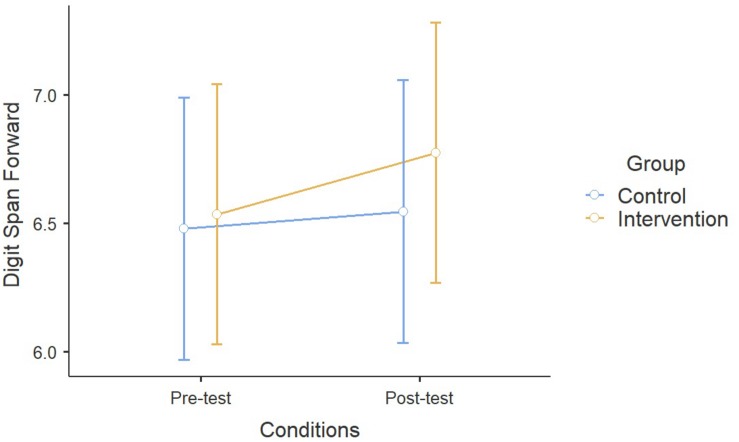
Results on the Digit Span Forward.

**FIGURE 3:**
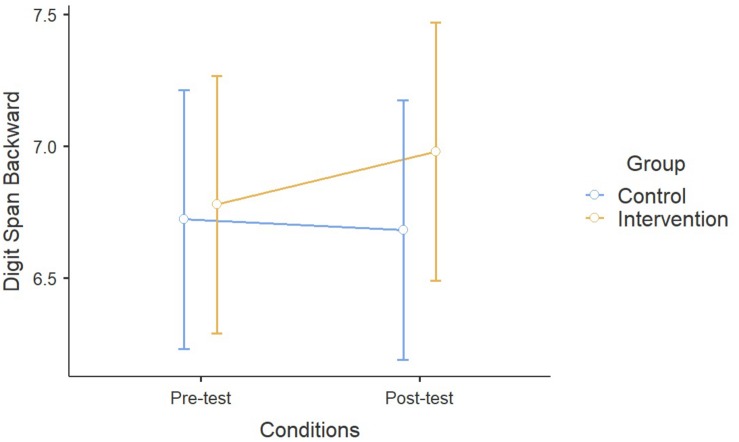
Results on the Digit Span Backward.

For what concerns Digit Span Forward, repeated measures ANOVA showed a significant effect of Time between pre- and post-test conditions, while non-significant effects were found in the interaction between Time and Activity (*F*_1_,_355_ = 2.26, *p* = 0.13). No general performance improvement was observed in the Backward recall during time (*F*_1_,_351_ = 0.21, *p* = 0.64), but children from the ESA Program significantly improved more their performance compared to the control group (*F*_1_,_351_ = 4.58, *p* < 0.05). No effects of Age were detected in both cases (Forward: *F*_1_,_355_ = 1.04, *p* = 0.31; Backward: *F*_1_,_351_ = 0.05, *p* = 0.83) (Figures 2, 3).

**FIGURE 4:**
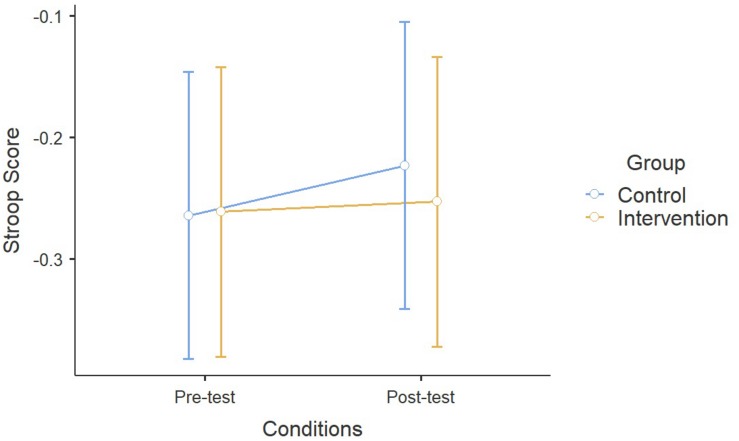
Results on the Stroop Task.

**FIGURE 5:**
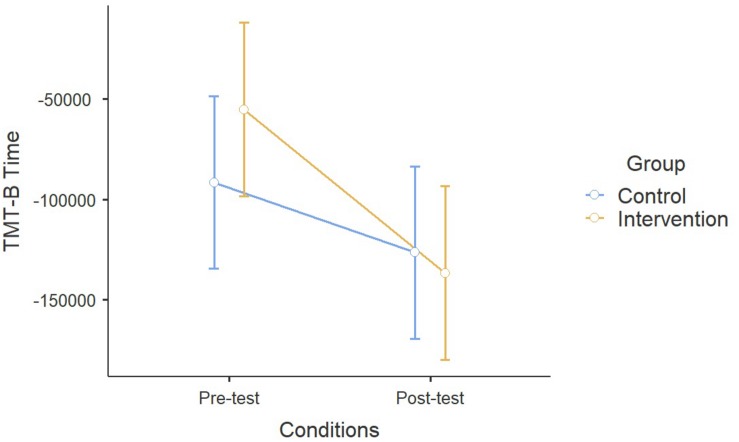
Results on the Trail Making Test.

A significant effect of Age has been found on the Stroop Task (*F*_1_,_338_ = 6.98, *p* < 0.01), but both the Time effect and the interaction between the treatment and the group were non-significant (Time: *F*_1_,_335_ = 2.34; *p* = 0.12; Time × Group: *F*_1_,_335_ = 0.87, *p* = 0.35) (Figure 4). Concerning the TMT, a significant effect of the ESA Program between pre-test and post-test conditions was found (*F*_1_,_348_ = 26.3, *p* < 0.00), as well as the interaction between Time and Group (*F*_1_,_348_ = 13.7, *p* < 0.001) and the influence of Age (*F*_1_,_348_ = 12.4, *p* < 0.001) (Figure 5).

## Discussion

The purpose of the current study was to test the effectiveness of the ESA Program on children’s cognitive performances, in particular, their EFs. The ESA Program is an enriched sports program containing warm-up physical exercises that were modified to stimulate three core EFs (inhibitory control, working memory, and task shifting) (Diamond, 2013).

Some studies have already proven that the introduction of an ESA program enhances cognitive functioning (Beck et al., 2016; Gheysen et al., 2018). In this study, we specifically tested changes in working memory and short-term memory through Digit Span Forward and Backward Task, inhibitory control through the Stroop Task, cognitive flexibility and task shifting through the TMT that are EFs involved in daily life and relating to school performances (Visu-Petra et al., 2011).

Moreover, the ESA Program has already shown its effect concerning children’s physical fitness, where moderate effects were found in relation to throwing, jumping, sprinting, and agility (Duda et al., 1991). Our sample was made up of children coming from four European countries that implemented the ESA Program within the same period and following the same protocol, shared with all the partners through video tutorials. In this way, the standardization of the procedures was ensured.

For the evaluation of the program effectiveness, repeated measures ANOVA was run, revealing a significant effect of the ESA Program on Digit Span Backward and TMT B version. No significant effect of the ESA Program was found on Digit Span Forward nor on the Stroop Task. To summarize our results, the enriched sport program produced positive effects for working memory, task switching, and cognitive flexibility, while no beneficial effects were detected on inhibitory control and short-time memory. Concerning the age of the sample, a significant interaction with the ESA Program was found in the Stroop Task and TMT, indicating that older children significantly reduced the completion time of the task, but the same effect was not detected for Digit Span Forward and Backward.

The positive effect of an enriched sports program on working memory is in line with ample research. Both longitudinal and cross-sectional studies underlie a positive association among structured physical activity in childhood with higher working memory performance (López-Vicente et al., 2017). Koutsandreou et al. (2016) found that 10 weeks of interventions based on cardiovascular and motor afterschool exercise programs enhanced working memory skills on a sample of 9- to 10-year-old children with a larger degree due to the motor exercise intervention rather the cardiovascular program.

Moreover, a study by Beck et al. (2016) employed a sport-enriched math program made up of three conditions, namely, gross motor math group, fine motor math group, and control condition. The results showed that children from all the conditions improved their performance, but the ones from gross motor condition enhanced their performance significantly more than the other groups when tested immediately after the program. This enhancement was not found after 8 weeks.

The ESA Program’s effects on TMT B reduction of time is coherent with findings of a previous study carried out by Schmidt et al. (2016). The authors compared four experimental conditions characterized by an increasing cognitive load, ranging from low cognitive demand to high cognitive demand, and increasing physical demand. They found that following a period of 10 min of cognitive challenging tasks, children showed better focused attention and decreased processing speed. The speed component of the attention was highly influenced. These findings focus on the issue concerning the quality of intervention. Cognitive challenging physical activities revealed to be more suitable to improve children’s attention compared to ordinary physical activities.

No improvements of the ESA Program were found upon inhibitory control, a result that contradicts other data in literature (Tsai, 2009; Drollette et al., 2014). However, the studies that found a positive effect of physical exercise on inhibitory control used clinical samples including children with specific problems, like ADHD (Chang et al., 2014), while our sample was made up of children from ordinary school class. It seems that children with poorer performance on EF tasks may experience more positive effects following sports programs compared to children exhibiting baseline normal performance on EFs (Ludyga et al., 2016).

The review by van der Fels et al. (2015) pointed out the interplay between cognitive and motor skills in childhood. The authors emphasized how challenging tasks stimulate the co-activation of prefrontal cortex, cerebellum, and basal ganglia and trigger common processes as inhibition, planning, and monitoring. The current study introduces many advantages, as the combination of different sport movement domains and cognitive stimulation that provide to the coaches an innovative structured warm-up characterized by an increasing cognitive load. A higher cognitive load task requires higher attention and regular physiological condition and muscular fatigue may impair cognitive performance. For this reason, the warm-up phase has been selected as the most appropriate exercise unit section for a suitable cognitive stimulation. Moreover, the physical assessment provided through the first phase of the Program has demonstrated its effectiveness also from a physical point of view (Thomas et al., 2020).

Some limitations of the present study should be noted. First of all, follow-up measurements were not implemented; thus, we do not have information about the stability of our results during time. Secondly, performances in EFs were detected, but an important limitation is that they were not linked to children’s academic achievement or their reading, writing, and calculating skills. Finally, construct validity was only assessed qualitatively. Given these limitations, future research should investigate the effects of enriched sport programs with accurate construct validity measures on children’s academic achievement with a follow-up period after the conclusion of the program.

Providing enriched programs, as the one suggested by the ESA Program, at school may significantly improve children’s cognitive functioning that in turn should have beneficial effects on their academic performances.

## Data Availability Statement

The raw data supporting the conclusions of this manuscript will be made available by the authors, without undue reservation, to any qualified researcher.

## Ethics Statement

The studies involving human participants were reviewed and approved by Lithuanian Sports University’s Research Ethics Committee in Social Sciences. Written informed consent to participate in this study was provided by the participants’ legal guardian/next of kin.

## Author Contributions

AG, AB, and MA contributed to the conceptualization. FŞ, ÖG, SP, VI, YD, DS, AB, and MA contributed to the data curation. AG, SB, and MG-L contributed to the formal analysis. AB and MA contributed to the funding acquisition and contributed to the project administration. FŞ, ÖG, SP, VI, YD, DS, and MG-L contributed to the investigation. AG, MA, YD, DS, and MG-L contributed to the methodology. SP, VI, and MG-L contributed to the resources. FŞ and ÖG contributed to the software. SP, VI, and AB contributed to the supervision. YD and DS contributed to the validation. AG and MA contributed to the visualization. AG, SB, AB, MA, and MG-L contributed to the roles/writing of the original draft. FŞ, ÖG, SP, VI, YD, and DS contributed to the writing, reviewing, and editing.

## Conflict of Interest

The authors declare that the research was conducted in the absence of any commercial or financial relationships that could be construed as a potential conflict of interest.
